# Socioeconomic and demographic differentials in unintended pregnancies, abortion, and contraceptive use in Spain: insights from the 2018 Fertility Survey

**DOI:** 10.1186/s12978-026-02329-6

**Published:** 2026-04-14

**Authors:** Marta Seiz, Xiana Bueno, Evgeniya Borisova

**Affiliations:** 1 Institute of Economics, Geography and Demography, Spanish National Research Council (CSIC), c/Albasanz 26-28, 3E19, Madrid, 28037 Spain; 2 School of Public Health, Department of Applied Health Science, Indiana University, Bloomington, IN USA; 3 Barcelona, Spain

**Keywords:** Social inequality, Unintended pregnancy, Abortion, Contraception, Reproductive health

## Abstract

**Background:**

There is growing interest in the social stratification of demographic trends, including those related to reproductive outcomes. One issue that has received little attention in recent years in the case of Spain is the relationship between individuals’ social position and unintended pregnancies, abortion, and contraceptive use.

**Method:**

We present descriptive statistics and estimate predicted probabilities from logistic regression models using a nationally representative sample of 14,556 women from the 2018 Spanish Fertility Survey.

**Results:**

Results show that unintended pregnancies are more common among socially vulnerable women—those with a lower level of education, migrant background (not born in Spain), unstable partnership histories, or experience of employment instability. Abortions are also more prevalent within these groups. In contrast, women with higher socioeconomic status are more likely to avoid unintended pregnancies through more consistent contraceptive use.

**Conclusion:**

These findings highlight persistent social inequalities in reproductive health outcomes in Spain, suggesting the need for policy interventions that include educational programs on family planning and expand accessibility to reproductive health services particularly for the most vulnerable populations.

**Supplementary Information:**

The online version contains supplementary material available at 10.1186/s12978-026-02329-6.

## Abstract (plain language summary)

Using data from the 2018 Fertility Survey, this study looks at which social groups in Spain are more likely to experience unintended pregnancies, to undergo abortions, and to use birth control. It finds that unintended pregnancies are more common among women who are less educated, who were born abroad, who have been in unstable relationships or who have experienced job instability. Abortions are also more likely among these women. Women with higher education, who are employed, born in Spain and married tend to avoid unintended pregnancies by using birth control more consistently. These results show ongoing inequalities in reproductive health in Spain and point to the need for public policies that improve access to information and reproductive health services—especially for the most vulnerable groups.

**Supplementary Information:**

The online version contains supplementary material available at 10.1186/s12978-026-02329-6.

## Non-English language abstract (Spanish)

Existe un creciente interés por la estratificación social de las tendencias demográficas, incluidas aquellas relacionadas con las trayectorias reproductivas. Una cuestión que ha recibido escasa atención en los últimos años, en el caso de España, es la relación entre la posición social de los individuos y los embarazos no planificados, el aborto y el uso de anticonceptivos. Este estudio presenta estadísticas descriptivas y estima probabilidades predichas a partir de modelos de regresión logística utilizando una muestra representativa de 14.556 mujeres de la Encuesta de Fecundidad de 2018. Los resultados muestran que los embarazos no planificados son más comunes entre las mujeres socialmente vulnerables, como aquellas con menor nivel educativo, origen migrante (no nacidas en España) o con trayectorias de pareja o laborales caracterizadas por la inestabilidad. Los abortos son también más prevalentes en estos grupos. En cambio, las mujeres con un nivel socioeconómico más alto son más proclives a evitar embarazos no planificados mediante un uso más consistente de métodos anticonceptivos. Estos resultados ponen de manifiesto desigualdades persistentes en los resultados de salud reproductiva en España y sugieren la necesidad de intervenciones políticas que incluyan programas educativos relativos a la planificación familiar y una mayor accesibilidad a los servicios de salud reproductiva, especialmente para las poblaciones más vulnerables.

## Background

Understanding women’s characteristics behind the occurrence of unintended pregnancies, the decision to undertake an abortion, and the use of contraception is essential for advancing women’s reproductive health and overall well-being. These aspects of reproductive health not only shape individual life trajectories but also have broader social and economic implications. On this point, research consistently shows that unintended pregnancies are linked to adverse health and socioeconomic outcomes (see [1, 2]), with access to family planning services and reproductive healthcare playing a crucial role in mitigating these risks. Socioeconomic disparities in this regard can nonetheless be expected, as structural barriers to reproductive care, including financial constraints, geographic inaccessibility, lack of information, and restrictive policies, disproportionately affect marginalized populations [3–5], further exacerbating health and economic inequalities. Examining the relations between the characteristics of population groups and their reproductive outcomes is critical for informing policies and interventions that promote reproductive autonomy and health equity.

While there is evidence from postindustrial societies indicating that individual socioeconomic resources and demographic characteristics affect contraceptive use, unintended childbearing, and the decision to terminate or complete a pregnancy (see for example [2, 6]), this study focuses on Spain, a country where trends in unintended pregnancies, abortion, and contraceptive use have not yet been systematically analyzed using recent, representative, national-level data. The Spanish case is interesting, moreover, against the background of a universal, high-quality healthcare system which nonetheless has shown access barriers for certain population groups [7, 8], large temporal and geographical variability regarding financing of contraception [9], and successive reforms of abortion legislation [10]. Drawing on a nationally representative fertility survey from 2018, this study examines whether outcomes related to unintended pregnancies, abortion, and contraceptive use align with a continuum of social disadvantages that may limit access to reproductive care. First, we assess whether individuals in more vulnerable situations, defined by socioeconomic and demographic factors such as education, migrant background, age, partnership trajectories, or employment, are more likely to experience unintended pregnancies. We also examine whether they have a higher probability of abortion. Second, we explore the relationship between socioeconomic disadvantages and the use of contraception and effective methods, to see whether unintended pregnancy and abortion outcomes in Spain reflect birth control patterns.

### Contraceptive use, unintended pregnancies, and abortion

The relationship between contraceptive use, unintended pregnancies, and abortion is intricate. While higher rates of unintended pregnancies among disadvantaged populations are often attributed to limited access to contraception, this trend persists even in settings where contraception is provided free of charge. This suggests that factors such as knowledge gaps regarding proper use, access barriers, and cultural attitudes fostering ambivalence or reluctance toward contraception play a significant role [1]. Likewise, although contraception generally reduces abortion rates, some countries with widespread contraceptive access still report a high frequency of abortion, a pattern linked to contexts experiencing rapid fertility decline [11]. Another layer of complexity is that contraceptive use appears to be more influenced by relationship dynamics than by socioeconomic status [2].

It is estimated that 44% of all pregnancies around the world are unintended [12, 13]. In low- and medium-income countries, unintended pregnancy is strongly linked to limited access to contraceptives (especially safe and modern ones), to ineffective contraceptive use, and to the unavailability of health and family planning services that can provide information and help women to prevent unintended pregnancies [14]. In high-income countries, studies show that unintended pregnancies are generally more prevalent among young, unpartnered women with lower education levels [2], low income [1, 6], or migrant backgrounds, particularly undocumented migrants [15]. Contributing factors mainly include restricted access to contraception, inadequate knowledge about use, difficulties in adherence, and irregular gynecological visits [1, 16]. Additionally, migrants in certain contexts face restricted access to reproductive care due to policy barriers, discrimination, lack of healthcare access, insufficient information, or social isolation [6]. For some people, more complex factors have also been claimed to intervene, such as ambivalences towards childbearing, a lack of perception of pregnancy as something than can/is to be planned, feelings of little control over one’s reproductive capacity, or situations where the individual is neither trying to achieve a pregnancy nor to prevent it [17].

Regarding abortion, multiple indicators of social disadvantage, such as young age, being single or in an unstable relationship when conceiving, lower education levels, financial insecurity, and migrant background, are consistently associated with higher abortion rates across Western societies [2, 18–21]. However, in some countries, such as the United States, the high financial costs of abortion increase the likelihood that socially vulnerable individuals carry unintended pregnancies to term [22]. In many low-and middle-income countries, on the other hand, there is no legal right to abortion or access is highly restricted [23, 24], and many women resort to medically unsafe abortion methods [25]. In many of these societies, accordingly, abortion is more common among women with higher socioeconomic status, who tend to have greater motivation and resources to realize their fertility preferences. At the same time, these women usually have greater access to information and to healthcare services, so they are less likely to resort to medically unsafe abortion methods than their more socioeconomically disadvantaged counterparts [26].

### The Spanish context

Spain presents a compelling case study for analyzing the linkages between socioeconomic and demographic characteristics and women’s reproductive health. Traditionally a Catholic country, Spain has undergone rapid secularization and significant shifts in gender-role norms [27]. Notable changes include a reversal in the gender gap in education [28], a substantial increase in women’s labor force participation [29] and relevant intergenerational changes in gender norms surrounding reproductive behavior [30]. These transformations have contributed to generally advancing women’s reproductive autonomy. However, socioeconomic disparities can still be expected to shape reproductive health outcomes. Attitudinal and behavioral sociodemographic transformations have namely not been uniform across the population, as witnessed by a significant plurality of family trajectories, patterns, and forms [31]. Furthermore, the accessibility of contraception and abortion has varied over time and across regions. It was first in 2010 that universal access to reproductive healthcare services and programs and to safe, efficacious contraceptives was legally protected through the Organic Law 2/2010 on Sexual and Reproductive Health and the Voluntary Interruption of Pregnancy [32]. This law also established that access to abortion up to 14 weeks without restrictions would be guaranteed. Nevertheless, difficulties and heterogeneity in the application of norms and policies, resulting in variations in access to different services, have been identified ever since [33] .

The extent to which gaps in access to reproductive care are mirrored by socioeconomic and demographic differentials in unintended pregnancy, recourse to abortion, and contraceptive use is a question that remains underexplored due to limited data availability. The 2018 Spanish Fertility Survey [34], which provides representative data on fertility behavior and relevant social variables, offers a valuable opportunity to address this research gap at the national level.

Some earlier studies have found higher unintended pregnancy rates among women with lower education levels and overall lower socioeconomic status [35, 36]. Women from disadvantaged backgrounds have also been observed to opt more frequently for abortion when facing unintended pregnancies [35, 36]. Abortion rates have proven particularly high among women with lower education levels [37], which has recently been confirmed to be the case with census data and regardless of age [38]. Given that Spain has one of the latest mean ages at first childbirth worldwide, it is unsurprising that abortion rates are highest among women under 25 compared to those aged 25–34 [37, 39–41].

Certain groups of immigrant women in Spain have been noted to face significant barriers to family planning services due to economic constraints, bureaucratic obstacles, and lack of information [36]. Single and very young women have also encountered accessibility challenges [35]. Consequently, women with migrant backgrounds, especially those from Sub-Saharan Africa and Latin America, have been found to resort to abortion more frequently than Spanish-born women; a behavior often linked to precarious employment [36, 38, 42]. Beyond individual characteristics, contextual factors also play a role—regions with lower public expenditure, no local universities, and higher levels of immigration tend to show higher abortion rates [37].

Women from lower socioeconomic backgrounds in Spain have also reported difficulties accessing healthcare services and using contraception regularly, particularly in more deprived areas [43, 44]. At the same time, they have been found to be more likely to engage in unprotected sex and to experience higher rates of contraceptive failure [45]. Notably, it has been shown that a significant proportion of women seeking abortions were already using some form of contraception, suggesting inconsistent or intermittent use [46]. However, in their study, Lete et al. [47] also found that among the 31% of women who did not use contraception, 21% were sexually active and did not wish to become pregnant, thereby making them vulnerable to unintended pregnancies. Meanwhile, prior abortion experiences have been shown to increase the likelihood of switching to more reliable hormonal contraceptive methods [48]. Disparities in contraceptive use are also evident among immigrant women, with Sub-Saharan women reporting the lowest rates [49]. Additionally, Eastern European and Latin American immigrant women in Spain often become mothers earlier than desired due to unintended pregnancies [50].

### Current study and hypotheses

In summary, previous research on Spain points at socioeconomic differentials in the risk of (1) experiencing unintended pregnancy (2), undergoing an abortion, and (3) failing to use contraception. There is a pressing need, nevertheless, for updated analyses that confirm, using nationally representative data, whether there is an association between these reproductive outcomes and various factors of social vulnerability. This is the purpose of our study, which ultimately also seeks to establish whether reproductive outcomes choices related to unintended pregnancies align with differences across the socioeconomic spectrum, particularly by education, migrant origin, income, or labor market attachment.

In line with prior findings, we propose the following hypotheses:

- *Hypothesis 1: The most vulnerable women – e.g., younger women, those with lower educational level, those with a migrant background*^1^*, or those with unstable partnership and employment circumstances – will be more likely to have experienced unintended pregnancies*

This expectation rests on the noted reproductive care accessibility challenges observed in previous research for certain population groups who could have limited information on how to locate, navigate, and use family planning services. We also expect women having experienced partnership instability to be more exposed to unintended pregnancies due to potentially less consistent use of the most effective contraceptives (such as hormonal methods). Women with unstable employment circumstances could have had difficulties to economically access contraception.

- *Hypothesis 2: We expect women in relatively disadvantaged social positions and in conditions of economic insecurity, yet not in the most vulnerable population segments, to be more likely to have experienced abortion. We also expect abortion to be more prevalent among women with a history of partnership instability.*

The expected relation between a higher probability of having undergone abortion and a position of relative social disadvantage is largely built on the same assumption than H1 – these women will likely be more exposed to unintended pregnancies. Furthermore, we expect them to have limited economic and social resources to face childbearing from unintended pregnancy. Nevertheless, given the previously noted gaps in universality of access to publicly financed abortion, moderately disadvantaged women with a certain degree of socioeconomic and/or information resources (i.e. those in employment even if precarious, those with medium-level education) could be more likely to interrupt their pregnancies than those in greatest social vulnerability. Women having experienced greater partnership instability could also be more likely to have undergone abortion, both due to possibly less consistent contraception patterns and to the lack of partner support to face unexpected childbearing.

- *Hypothesis 3: Women with higher socioeconomic status, particularly those with higher educational attainment, will be more likely to prevent unintended pregnancies through more consistent use of contraception and the choice of more effective methods.*

We propose, thus, that women in more advantaged social positions will manage to avoid unintended pregnancies and thus also show lower abortion probabilities through higher rates of use of contraception and access to the most efficacious methods. Factors possibly facilitating such behavior would be greater economic capacity, potentially greater awareness of available methods and reliable use; and easier access to family planning and healthcare services that can provide counseling.

## Methods

This study utilizes data from the 2018 Spanish Fertility Survey [34]. This cross-sectional survey, which also includes retrospective information on key variables, contains a nationally representative sample of 14,556 women aged 18–55 years old. It provides both current (referring to the time of the interview) and recalled data on several fertility-related dimensions, as well as on a wide array of demographic, economic, and social characteristics of respondents.

After a first overview of the sampled women’s characteristics, we present descriptives of the prevalence of unintended pregnancies, abortion, and contraceptive use across different categories of key socioeconomic and demographic indicators^2^: educational level (primary or less / secondary and post-secondary / university); migrant origin (Spanish born / foreign born); birth cohort (1960s / 1970s / 1980s / and 1990 and year 2000); number of co-residential unions as a proxy for partnership (in)stability (categorical variable from 0 to 3+)^3^; partnership status at the time of the survey (married / in a registered partnership / in non-registered cohabitation / in a non-coresidential relationship / single); number of jobs as a proxy for employment (in)stability (categorical variable taking the values 0, 1 to 3, 4 to 6, and 7+ )^4^; employment status at the time of the survey (employed / non-employed); individual net monthly income at the time of the survey (no income / less than 1000 euros/ 1000–1999 euros / 2000–2999 euros / more than 3000 euros); religiosity^5^ (little or no practice / quite frequent or very frequent); and place of residence (rural / non-rural). This set of variables represents the core factors considered in our models when potentially relevant for the analyzed outcome.

We have conducted three sets of models, one for each outcome variable (i.e., unintended pregnancies, abortion, and contraceptive use). We have run binary logistic regression models with heteroscedasticity-consistent robust standard errors. All models, which are presented as supplementary material in the Appendix (Models 1 to 8), report odds ratios with a 95% confidence level. They constitute the basis for the estimation of average predicted probabilities (predictive margins) of each outcome for different categories of the independent variables, which are presented in the results section. We have chosen to report and graphically present^6^ average predicted probabilities rather than odds ratios in the main text to ease the interpretation of results, since predicted probabilities provide a more intuitive depiction of the magnitude of the associations observed. When graphically representing predicted probabilities, we focus on five types of variables that we consider to be key socioeconomic and demographic indicators: educational level, migrant origin, birth cohort or age, partnership trajectory or situation, and employment trajectory or situation. Results corresponding to control variables are only discussed when relevant along the text. Next, we describe the rationale behind the selected models, the outcomes, and variations in the inclusion of covariates.

### Measurement and analysis of unintended pregnancies

After asking whether the respondent was or had ever been pregnant and how many times, the 2018 Spanish Fertility Survey asked participants, *‘Was it in your plans to have a baby/ Have all your pregnancies been planned?’* with response options of ‘Yes / No’. We use these measures to analyze whether it is more likely for unintended pregnancies to occur, as expected in hypothesis H1, among women in the most vulnerable situations – specifically, younger women, those with a lower level of education, those with a migrant background, those in unstable partnerships, and those having experienced employment instability. To this end, we first estimate a regression model using a binary indicator of unintended pregnancy as the outcome (value 1 = the respondent has experienced an unintended pregnancy; value 0 = the respondent has not experienced an unintended pregnancy). The model includes maternal education, migrant origin, birth cohort, number of partnerships, and number of jobs as covariates, as well as religiosity and rural residence as control variables. We have estimated the model both on the total sample of women (*n* = 11,290 after removing respondents with missing values, Model 1 in the Appendix) and – as a robustness check – on a subsample of women having experienced at least one pregnancy (*n* = 6,692, Model 2 in the Appendix). This is done to make sure that results are generally observable and not driven by the composition of the sample and aspects related to selection into parenthood/non-parenthood.

Additionally, we assess the predictive value of socioeconomic and demographic characteristics on the likelihood of having had a first birth from an unplanned conception (*n* = 1,312, Model 3 in the Appendix) among women having had only one pregnancy. The dataset provides information on whether the respondent has experienced an unintended pregnancy in the past, yet it is not possible to trace it back to a specific gestation. Accordingly, to know for sure whether a given pregnancy was intended or not and to include covariates that are potentially concurrent in time to the different intended and unintended pregnancies, we must limit this part of the analysis to women having undergone just one pregnancy. The outcome for Model 3 is a dichotomous variable indicating whether a woman’s first birth resulted from an unintended (1) or intended (0) pregnancy. The above-mentioned covariates and controls are included, with education, employment (employed versus non-employed), age (instead of birth cohort)^7^ and partnership situation (had a partner versus partnerless) measured at the time (year) of the first birth (instead of at the time of the survey, in the case of education, or in terms of stability, in the case of partnership and employment situation, as was done in previous models). This model provides insights on the characteristics of women who not only have had unplanned pregnancies but also have completed them. More specifically, it allows us to examine the factors associated with births resulting from such pregnancies as opposed to those resulting from planned conceptions.

### Measurement and analysis of abortion

After asking whether the respondent had ever been pregnant and how many times, the 2018 Spanish Fertility Survey posed the questions: “*Was any of them a failed pregnancy (miscarriage or abortion)* and, as a follow-up, *“Was any of the pregnancies a miscarriage”?* The two questions, which could be answered with ‘Yes’ or ‘No’, make it possible to identify women who have had at least an abortion and no miscarriages. Nevertheless, they do not allow us to capture potential abortions of women who have had several failed pregnancies with at least one of them being a miscarriage. These individuals are namely automatically classified as having had spontaneous pregnancy interruptions, without any information on additional voluntary ones being provided. This limitation of the dataset suggests that we might be underestimating somewhat the extent to which the sampled women have experienced abortions.

To test our second hypothesis, we examine the probability of having undergone an abortion in relation to potentially relevant socioeconomic and demographic characteristics in the total sample of women (*n* = 13,129 after removing respondents with missing values, Model 4 in the Appendix). We incorporate the main set of covariates included in the previous stage of the analysis (models 1 and 2)^8^.We find it important to include in the regression women who have never been pregnant, since they are likely to capture, to a substantial extent, adequate access to/use of effective contraception, which must be considered when assessing the probability of abortion. As a robustness check, nevertheless, we also run the model only on women who have experienced a pregnancy (*n* = 7,824, Model 5 in the Appendix).

### Measurement and analysis of contraceptive use

The survey asked participants, ‘*Are you or your partner using any type of contraception?*’ with ‘Yes’ and ‘No’ as response options. This question represents our dichotomous dependent variable – use of contraception versus non-use – in the third set of models. The use of contraception contemplates the following options: hormonal contraception (birth control pills, vaginal ring, transdermal contraceptive patch, hormonal intrauterine devices, injectable contraceptives, emergency contraception pills), copper intrauterine devices, barrier methods (male and female condom, diaphragm, cervical cap/ sponge, spermicides), surgical sterilization (tubal ligation, vasectomy), and other methods (including those based on periodical abstinence or withdrawal).

In these models, we test the third hypothesis and explore whether the expected lower prevalence of unintended pregnancies and abortion among women in more advantaged social positions could be mirrored by higher rates of contraceptive use as well as by access to more effective methods. For this purpose, we first assess the relation between contraceptive use and different socioeconomic and demographic indicators among women who were sexually active at the time of the survey, who were not pregnant, who did not desire to have children, and who were not biologically infertile (*n* = 12,368, Model 6 in the Appendix). Second, to assess potential socioeconomic and demographic differentials regarding the use of highly effective contraceptive methods, Model 7 (see the Appendix) explores whether respondents use a contraceptive method with a Pearl Index (PI)^9^ equal to/below 0.3 (value 1) [58] or not (value 0, which includes using a method lying above the noted threshold or not using contraception). Third, as a robustness check, the same regression model is performed only on women who reported using contraception (Model 8 in the Appendix), to test whether the findings hold or, on the contrary, seem to be driven by the sample’s composition.

The independent variables in Models 6–8 include some of the previously described sociodemographic characteristics (i.e., education, migrant background, religiosity, and place of residence), as well as new covariates variables: age (18–24 / 25–29 / 30–34 / 35–39/ 40–44/ 45 + years old), partnership status (singlehood / non-coresidential relationships / unregistered cohabitation / registered partnerships / marriage), employment status (employed / not employed), and individual net monthly income (no income / less than 1000 euros / between 1000 and 1999 euros / between 2000 and 2999 euros / more than 3000 euros).

## Results

### Descriptives

Sample descriptives showing the distribution of observations across the different dependent and independent variables’ categories are provided in Table 1. Almost 19% of women aged between 18 and 55 years old in 2018 had experienced at least one unintended pregnancy according to the Spanish Fertility Survey. The percentage of women having undergone at least one abortion was 3.6%, and only 55.3% of the sample were using contraception.

**Table 1:** Sample descriptives

All sampled women	*n*	%
Total	14,556	100
*Unintended pregnancies*		
Has experienced at least one unintended pregnancy	2,356	16.2
Has never experienced an unintended pregnancy	10,136	69.6
Missing	2,064	14.2^a^
*Abortion*		
Has undergone at least one abortion	519	3.6
Has never undergone an abortion	14,037	96.4
*Contraceptive use at the time of the survey*		
Using contraception	8,053	55.3
Not using contraception	6,503	44.6
*Education at the time of the survey*		
Primary	1,903	13.1
Secondary and post-secondary	7,905	54.3
University	4,748	32.6
*Migrant origin*		
Spanish-born	12,808	88
Foreign-born	1,748	12
*Birth cohort*		
1960s	3,737	25.7
1970s	4,833	33.2
1980s	3,243	22.3
1990s+	2,743	18.8
*No of unions*		
0	2,527	17.4
1	10,622	73
2	1,217	8.4
More than 3	189	1.3
*Partnership status at the time of the survey*		
Married	7,084	49
Registered partnership	299	2.1
Non-registered cohabitation	1,401	9.7
In a non-coresidential relationship	1,909	13.2
Single	3,764	26
*No of jobs*		
0	1,861	12.8
1 to 3	4,635	31.8
4 to 6	3,560	24.5
More than 7	4,500	30.9
*Employment at the time of the survey*		
Employed	9,182	63.1
Not employed	5,374	36.9
*Individual net income at the time of the survey*		
No income	3,824	26.3
Less than 1000 euros	5,699	39.2
Between 1000 and 1999 euros	4,207	28.9
Between 2000 and 2999 euros	687	4.7
More than 3000 euros	139	0.9
*Religiosity*		
Practicing	11,488	78.9
Non-practicing	1,642	11.3
Missing	1,426	9.8
*Residence at the time of the survey*		
Urban	12,008	82.5
Rural	2,548	17.5

Data source: Spanish Fertility Survey (Encuesta de Fecundidad), 2018. Women aged 18-55 years old

^a^ The high percentage of missing values derives from a survey item inquiring whether the person was pregnant at the time of the interview, which was used in the construction of the unintended pregnancy variable. Unfortunately, no explanation is provided in the dataset as to the reason for the relatively high non-response rate found for this item. Despite this shortcoming, nevertheless, the Spanish Fertility Survey is still the most complete and reliable source of representative data on fertility available for Spain

Table 2 presents the percentage of individuals having experienced different outcomes (i.e., unintended pregnancy, abortion, and contraceptive use) by socioeconomic and demographic characteristics. Figures regarding *unintended pregnancies* and *abortion* are calculated for the total sample (*n* = 14,556) and excluding observations with missing values (see values of subsamples – n – in Table 1). Figures on *contraceptive use* draw on a more reduced sample (*n* = 12,368) because we considered it more pertinent to assess contraceptive use only among women at risk for unintended pregnancy that is, women who were sexually active by the time of the survey, who were potentially fertile, and who desired to avoid a pregnancy^10^.

**Table 2:** Proportion of women in each socioeconomic category having experienced the outcomes of interest

	Unintended pregnancy		Abortion	Contraceptive use	
	Unintended pregnancies (*n* = 12,492)	First birth from unintended pregnancy (*n* = 2,051) *	Abortion (*n* = 14,556)	Contraceptive use (*n* = 12,368)**	Contraceptive methods with P.I. >= 0.3 (*n* = 12,368)**
Total	18.9%	19.4%	3.6%	64.3%	28.7%
*Education*					
University	13.3%	11.4%	3.1%	71.8%	29.5%
Secondary and post-secondary	19.9%	23.1%	3.8%	64.2%	30.7%
Primary	30%	22.3%	3.8%	46.3%	18.7%
*Migrant origin*					
Spanish-born	17%	17.6%	3.2%	64.9%	28.8%
Foreign-born	31.9%	33.8%	6.5%	59.9%	27.3%
*Birth cohort*					
1960s	44.2%	21.2%	3%	37.6%	16%
1970s	19.6%	16%	4.3%	66.3%	27.3%
1980s	15.1%	18.7%	4%	78.3%	34.9%
1990s+	5.5%	47.1%	2.6%	82.3%	41.7%
*No of unions*					
0	5.6%	55.7%	1.4%	64.8%	28.6%
1	20%	16.7%	3.5%	64.6%	28.8%
2	33.9%	25.9%	7.2%	61.3%	28%
More than 3	46.8%	47.8%	14.8%	60.6%	24.4%
*Partnership status at survey time*					
Married	23.1%	13.1%	3.2%	59.7%	25.8%
Registered partnership	20.8%	20.3%	6%	71.9%	28.8%
Non-registered cohabitation	20.7%	21.3%	6.2%	71.3%	31.6%
Non-coresidential relationship	11.9%	36.8%	3.6%	80.8%	39.3%
Single	14.4%	36.8%	3%	60.8%	27.2%
*No of jobs*					
0	9.2%	23.9%	1.5%	64.4%	28.2%
1 to 3	18.5%	16.7%	2.8%	64%	28.9%
4 to 6	21.5%	20.1%	4.5%	65.4%	29.3%
More than 7	21.2%	21.2%	4.5%	63.8%	28.1%
*Employment at survey time*					
Employed	19.1%	17.9%	3.2%	65.7%	29.4%
Non-employed	18.5%	22.8%	3.8%	61.9%	27.4%
*Individual net income at survey time*					
No income	15.6%	22.5%	2.6%	64.1%	28.9%
Less than 1000 euros	22.4%	23.7%	4.2%	62.8%	28.3%
1000–1999 euros	17.1%	13.4%	3.6%	66.8%	29.6%
2000–2999 euros	20.2%	12.9%	3.9%	63.9%	25.7%
More than 3000 euros	9.6%	5.3%	2.2%	58.3%	20.8%
*Religiosity*					
Practicing	25.9%	23%	2.7%	55.2%	29.8%
Non-practicing	18.4%	18.9%	3.8%	66.1%	22.5%
*Residence*					
Urban	19%	19.9%	3.8%	64%	28.7%
Rural	18.9%	17%	2.7%	66%	28.5%

Data source: Spanish Fertility Survey (Encuesta de Fecundidad), 2018. Women aged 18–55 years old. Only subsamples with non-missing values in outcome variables.

*Women having undergone just one pregnancy ending in a birth; **Sexually active women who declare they do not wish to become pregnant and who are not biologically sterile

The descriptives anticipate that the occurrence of *unintended pregnancies* (in the general population) and the experience of a *first birth resulting from an unintended pregnancy* (among women having only experienced one pregnancy) are considerably more common among women with primary education or less (30% and 22.3%) than among those with university studies (13.3% and 11.4%). Women with non-university secondary and post-secondary education are also more prone to both phenomena (19.9% and 23.1%) than those with higher education (13.3% and 11.4%). The percentage of foreign-born women having experienced unintended pregnancies almost doubles that corresponding to Spanish-born women. There are thus certain socioeconomic gradients that already become apparent at the descriptive level regarding unintended pregnancy. Women with a migrant background and those with non-university studies have also undergone abortions to a greater extent (6.5% and 3.8%) than Spanish-born and highly educated women (3.2% and 3.1%). Abortion has also been more frequent among individuals with relatively unstable union trajectories.

The educational gradient and migration-related differences observed for unintended pregnancies and abortion are consistent with contraceptive use. Women with non-university education are visibly less prone to resorting to contraceptive methods when they reportedly want to avoid a pregnancy. Only 46.3% of women with primary-level studies or less and 64.2% of women with secondary and post-secondary non-university education use contraception, compared with 72% of women with higher education. Furthermore, low-educated women show particularly low rates of use of effective methods (hormone-based ones or vasectomy), not even reaching 19%. Women with high- and medium-level education do not exhibit high rates of use of such methods either, but they nonetheless approximate 30–31%. There are also gaps regarding contraception choices between Spanish-born and foreign-born women – the former show rates of use close to 65% when they intend to prevent a pregnancy, while the latter do not reach 60%. Differences in terms of contraception effectiveness are smaller, yet observable as well. Around 29% of Spanish-born women who need contraception resort to methods with a Pearl Index equal to or higher than 0.3, while the figure is lower, 27.3%, for foreign-born women. It is worth noting that, while contraceptive use among women who were at risk of pregnancy yet did not desire to conceive in 2018 was not itself high (64%), the recourse to provenly efficacious alternatives was particularly low (28.7%).

### Predictors of reproductive health outcomes

#### Unintended pregnancies

**Fig. 1:**
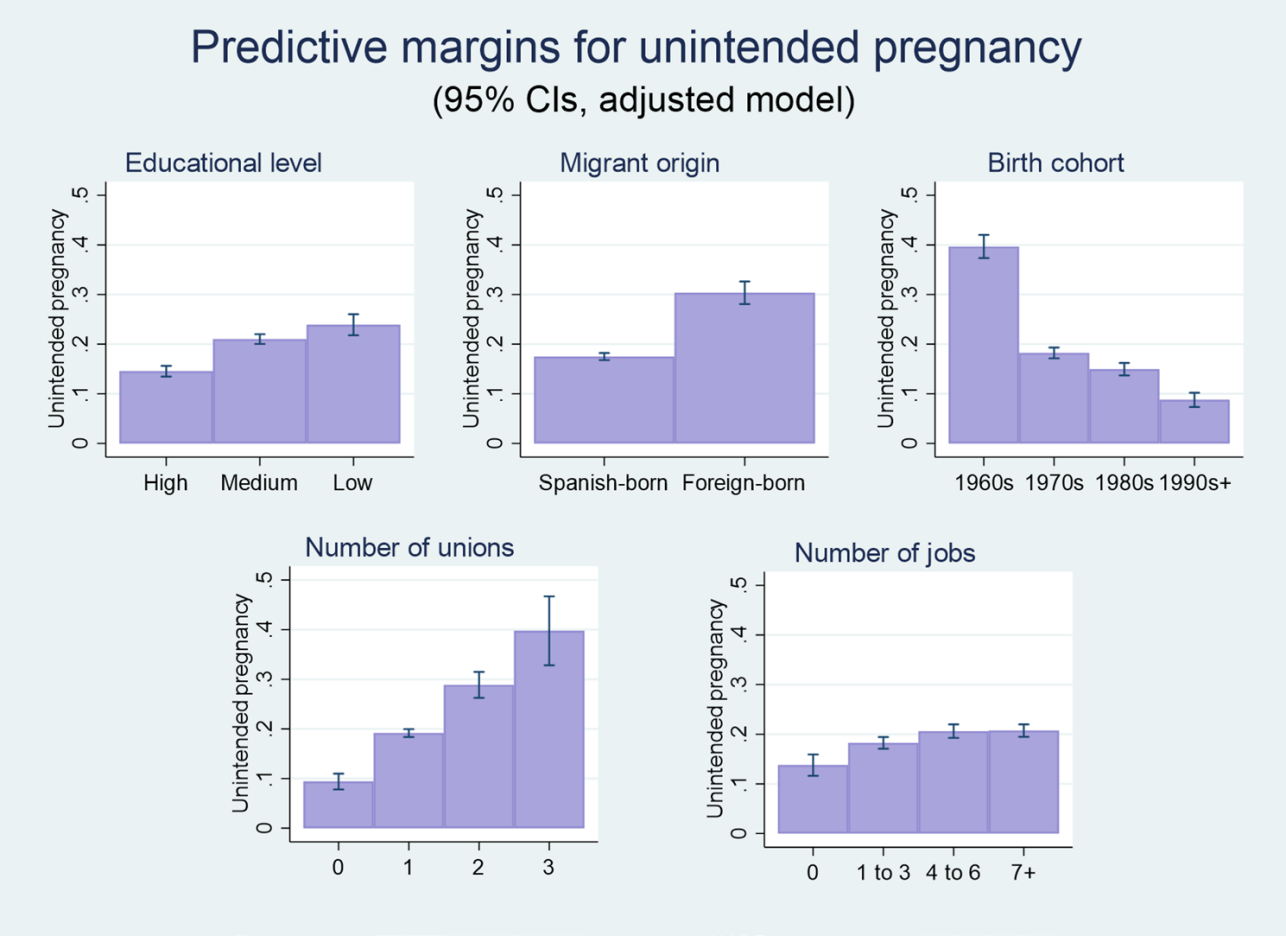
Average predicted probabilities of unintended pregnancy by socioeconomic and demographic variables (predictive margins as percentages). Data source: Spanish Fertility Survey (Encuesta de Fecundidad), 2018. Note: Results estimated from the adjusted binary logistic regression presented as Model 1 in the Appendix (Table A1). All sampled women aged 18–55 years old

The first set of logistic regression models assessed the likelihood of having had an unintended pregnancy. Results shown in Fig. 1 – derived from model 1 in Table A1, see the Appendix – indicate that foreign-born women in Spain are more likely to have experienced an unintended pregnancy at some point in their lives than native women. The average predicted probability of having undergone such a pregnancy amounts to 30.4% in the case of foreign-born women (95% CI: 0.28–0.33) and to 17.5% (95% CI: 0.17–0.18) in the case of Spanish-born women. Non-university education is also associated with a visibly higher likelihood of having experienced an unintended pregnancy. The average predicted probability is 23.9% (CI 95%: 0.22–0.26) for primary-level education or less; 21% (CI 95%: 0.20–0.22) for secondary or post-secondary level non-university education, and 14.6% (CI 95%: 0.13–0.16) for university-level education.

Additionally, the more co-residential unions the woman had been in, the higher her likelihood of having experienced an unintended pregnancy. In this case, the average predicted probability adds up to 39.8% for three or more unions (CI 95%: 0.33–0.47), to 28.9% for two unions (CI 95%: 0.26–0.31), to 19.2% for one union (CI 95%: 0.18–0.20), and to 9.4% for no unions (CI 95%: 0.08–0.11). Women who have had a relatively large number of jobs – adjusting for birth cohort – are also more likely to have had unintended pregnancies. The average predicted probability of this outcome is 20.7% for more than seven jobs (CI 95%: 0.19–0.22), 20.6% for four to six jobs (CI 95%: 0.19–0.22), and 18.3% for one to three jobs (CI 95%: 0.17–0.19), which can be compared to the 13.8% (CI 95%: 0.12–0.16) corresponding to women who had never been employed).

The likelihood of unintended pregnancy is lower for women in younger birth cohorts, especially as compared to those born in the 1960s, possibly due to both a shorter temporal exposure to the risk of pregnancy and more widespread use of effective contraception (the average predicted probability amounts to 8.9%, CI 95%: 0.07–0.10 for women born in the 1990s or 2000; to 15%, CI 95%: 0.14–0.16 for those born in the 1980s; to 18.2%, CI 95%: 0.17-19 for those born in the 1970s, and to 39.7%, CI 95%: 0.37–0.42 for those born in the 1960s).

Lastly, the religious practice control variable also bears a positive association with unintended pregnancy (for practicing individuals, the average predicted probability of this outcome is 23%, CI 95%: 0.21–0.25, which can be compared to the 18.7%, CI 95%: 0.18–0.19 corresponding to non-practicing individuals). These average predicted probabilities have been estimated from model 1, presented in Table A1 in the Appendix, where all estimated odds ratios underlying the noted relations are statistically significant at the 0.001 level.

As a robustness check, we run an additional model (Model 2) restricting the sample to women who have ever been pregnant. Results from Model 1 generally hold for Model 2 (in Table 3 in the Appendix), except for the number of unions, which no longer shows a positive association with unintended pregnancy.

**Fig. 2:**
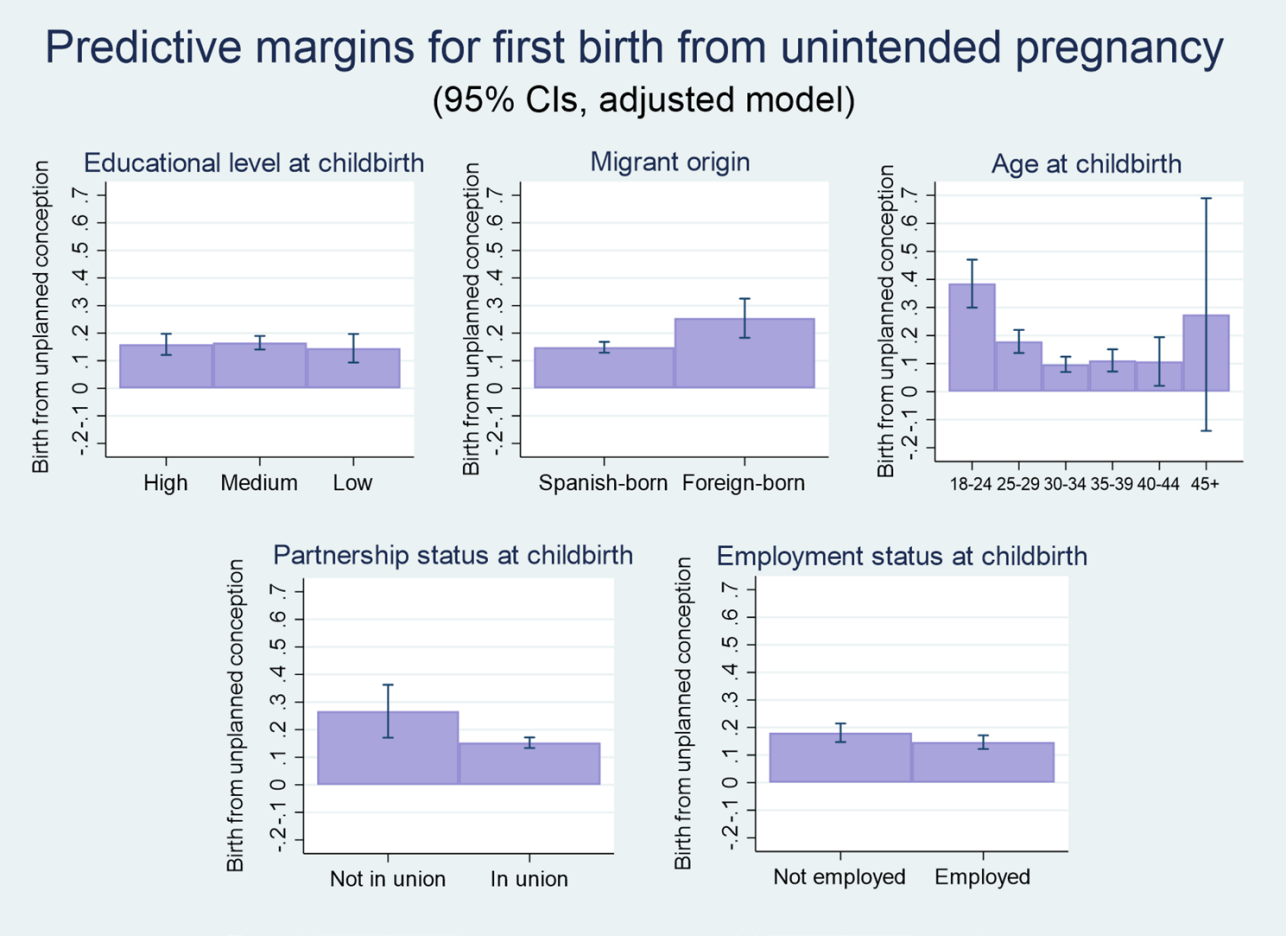
Average predicted probabilities of first birth resulting from an unintended pregnancy (predictive margins as percentages). Data source: Spanish Fertility Survey (Encuesta de Fecundidad), 2018. Note. Results estimated from the adjusted binary logistic regression presented as Model 3 in the Appendix (Table A2). Women aged 18–55 who have experienced a first birth

To further delve into the link between unintended pregnancy and socioeconomic disadvantages, we run a third model (Model 3 in Table A2 in the Appendix) restricting the sample to women who have been pregnant and given birth *once* to examine the factors associated with (first) births resulting from an unintended pregnancy (as opposed to those resulting from planned conceptions). In this case, covariates are included, whenever possible, based on the year the women became mothers. As observed in Fig. 2, educational attainment in the year of birth does not bear any significant relation to the outcome variable. The likelihood that a first birth is the result of an unintended pregnancy is greatest among women who conceived between ages 18 and 24, In this case, the average predicted probability if the noted outcome adds up to 38.5% (CI 95%: 0.30–0.47), a figure that is consistently higher than those observed for other age groups (17.9%, CI 95%: 0.14–0.22 for ages 25–29; 9.7%, CI 95%: 0.07–0.12 for ages 30–34; 11%, CI 95%: 0.07–0.15 for ages 35–39, 10.7%, CI 95%: 0.02–0.19 for ages 40–44; and 28%, CI 95%: -0.14-0.69 for age 45+). Foreign-born women also show a relatively high average predicted probability of unintended pregnancy (25.4%, CI 95%: 0.18–0.33, which can be compared to the 14.9% figure – C 95%: 0.13–0.17 – corresponding to women born in Spain).

The presence of a partner, in turn, is associated with a lower probability of having a first birth from an unintended pregnancy (average predicted probability of 15.3%, CI 95%: 0.13–0.17, which can be compared to the 26.7% CI 95%: 0.17–0.36 found for partnerless women). In the underlying logistic regression models presented in table A2 (see the Appendix) all relations are statistically significant at the 0.001 level, except for the negative association between having a partner and outcome of interest, which is significant at the 0.01 level. No statistically significant relations with maternal employment, religiosity or rural residence are found.

#### Abortion

**Fig. 3:**
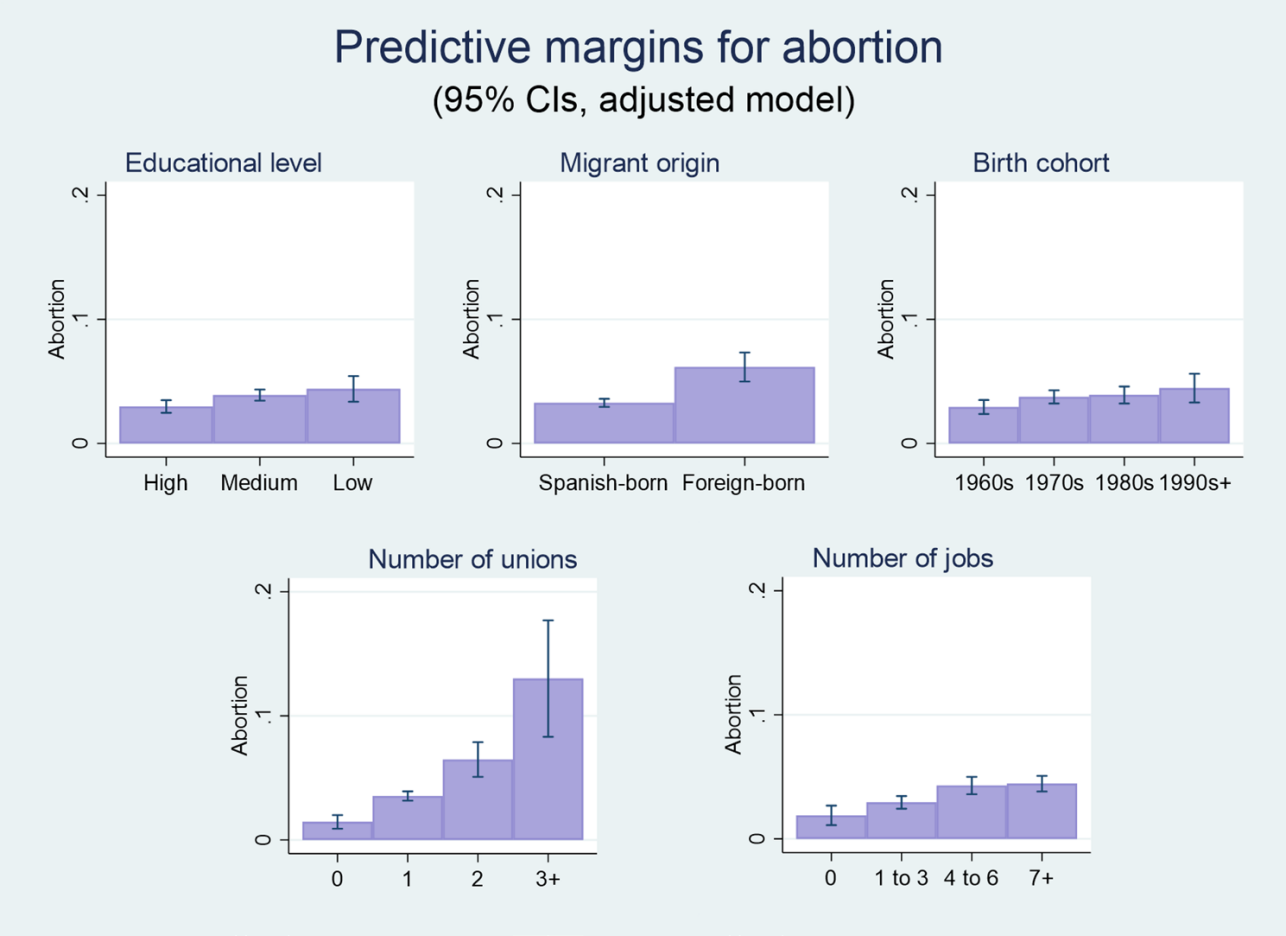
Average predicted probabilities of abortion (predictive margins as percentages). Data source: Spanish Fertility Survey (Encuesta de Fecundidad), 2018. Note: Results estimated from the adjusted binary logistic regression presented as Model 4 in the Appendix (Table A3). All sampled women aged 18–55 years old

The second set of logistic regression models assessed the likelihood of having had an abortion. Results shown in Fig. 3 (based on model 4, presented in the Appendix, Table A3) indicate that women without higher education are not only more likely to have experienced an unintended pregnancy, but also to have undergone an abortion, than women having completed university studies. The average predicted probability of this outcome is 4.4% for women with primary-level education or less (CI 95%: 0.03–0.05), 3.9% for those with medium-level education (CI 95%: 0.03–0.04) and 3% for women having completed university studies (CI 95%: 0.02–0.03). Foreign-born women are more prone to having had an abortion (average predicted probability of 6.2%, CI 95%: 0.05–0.07) than those born in Spain (average predicted probability of 3.3%, CI 95%: 0.03–0.04).

The probability of having had an abortion also increases with the number of co-residential partnerships. The average predicted probability of having experienced this event adds up to 13% (CI 95%: 0.08–0.18) for women having been in three or more unions, 6.5% (CI 95%: 0.05–0.08) for women having been in two unions, 3.5% (CI 95%: 0.03–0.04) for women having been in one union, and 1.5% (CI 95%: 0.01–0.02) for women who had never been in a union. In turn, individuals having had a relatively large number of jobs (more than three) and thus presumably having faced significant employment uncertainty are those most prone to abortion when employment histories are considered. The average predicted probability of this outcome amounts to 4.5% (CI 95%: 0.04–0.05) for women who had held seven jobs or more, to 4.3% (CI 95%: 0.04–0.05) for those having held between four and six jobs, to 2.9% (CI 95%: 0.02–0.03) for those who had held between one and three jobs, and to 1.9% (CI 95%: 0.01–0.03) for those who had never been employed.

Women born in the 1970s, 1980s, and, particularly, the 1990s (or 2000) are significantly more likely, when compared to respondents born in the 1960s, to have had an abortion. The average predicted probability estimated for the latter is 2.9% (CI 95%: 0.02–0.03), which can be compared to 3.8% (CI 95%: 0.03–0.04) for women born in the 1970s, 3.9% (CI 95%: 0.03–0.05) for women born in the 1980s, and 4.5% (CI 95%: 0.03–0.06) for women born in the 1990s or 2000. Individuals practicing a religion are less likely to have undergone an abortion (average predicted probability of 2.7%, CI 95%: 0.02–0.03) than non-practicing ones (average predicted probability of 3.8%, CI 95%: 0.03–0.04). Similar relations and virtually equivalent figures to those regarding religious practice are observed for women living in rural areas when compared to women living in urban areas. All noted relations in the underlying logistic regression model (see model 4 in table S3, Appendix) are statistically significant at the 0.05 level or below.

As in the previous section, we run a robustness check to confirm the validity of our results by running an additional model restricting the sample to women who have ever experienced a pregnancy. Model 5 in Table A shows that most associations previously observed in Model 4 hold in Model 5. Two observed changes between the two models are that differences across educational levels and employment categories are no longer statistically significant.

#### Contraceptive use

**Fig. 4:**
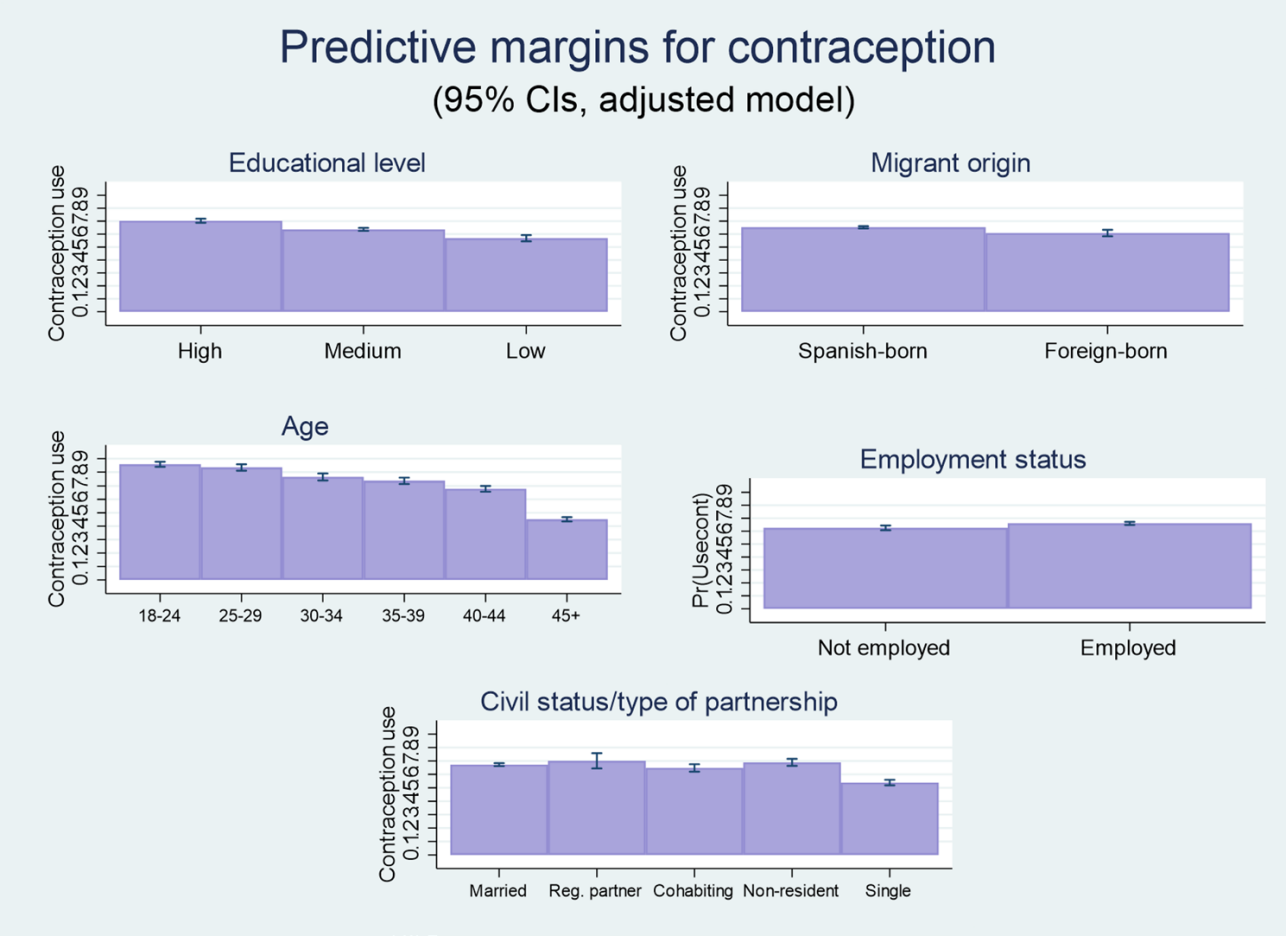
Average predicted probabilities of contraceptive use (predictive margins as percentages). Data source: Spanish Fertility Survey (Encuesta de Fecundidad), 2018. Note: Results estimated from the adjusted binary logistic regression presented as Model 6 in the Appendix (Table A4). Sampled women aged 18–55 years old who were sexually active, who were not pregnant or biologically infertile and who did not desire to conceive by the time of the survey

The third and last set of logistic regression models assessed the likelihood of using contraception among sexually active women who do not want to conceive. Results shown in Fig. 4 and in Table A4 (Model 6) in the Appendix indicate that the negative educational gradients observed in the probabilities of having an unintended pregnancy and an abortion hold, as hypothesized, a clear relation with contraceptive use. Thus, sexually active women with only primary-level studies or less are significantly less prone to using contraception when they do not desire a pregnancy (average probability of 56.8%, CI 95%: 0.54–0.59) than women with university studies (average probability of 70.3%, CI 95%: 0.69–0.72), as are women with secondary or post-secondary non-university education (average predicted probability of 63.6%, CI 95%: 0.62–0.65).

The likelihood of using contraception under the noted circumstances tends to decline gradually and significantly from age 30: while the average predicted probability of contraceptive use adds up to 85.8% (CI 95%: 0.84–0.88) at ages 18–24 and to 83.4% (CI 95%: 0.81–0.86) at ages 25–29, it goes down to 76.4% (CI 95%: 0.74–0.79) at ages 30–34, to 73.6% (CI: 0.71–0.76) at ages 35 to 39, to 67.8% (CI 95%: 0.66–0.70) at ages 40–44, and to only 45.1% (CI 95%: 0.44–0.47) at age 45 or above. Foreign-born women are less likely to use contraception (average predicted probability of 60.8%, CI 95%: 0.58–0.63) than Spanish-born women (average predicted probability of 65.3%, CI 95%: 0.64–0.66). Single women are less prone to use contraception to avoid an undesired pregnancy (average predicted probability of 53.9%, CI 95%: 0.52–0.56) when compared to married women (average predicted probability of 67.2%, CI 05%:0.67–0.68)^11^, while no statistically significant differences are observed (see Table A4, model 6, in the Appendix) between married individuals and those in any other kind of relationship.

Being employed, in turn, is associated with a higher probability of contraceptive use (average predicted probability of 65%, CI 95%: 0.65–0.67) than not being employed (average predicted probability of 62.5%, CI 95%: 0.61–0.64), but the relation with income is not statistically significant for any of the categories analyzed. Individuals who practice a religion are less likely to use contraception even if they declare they do not wish to become pregnant (average predicted probability of 59.5%, CI 95%: 0.57–0.62) than non-practicing ones (average predicted probability of 65%, CI 95%: 0.65–0.66), while women in rural areas also show a propensity for greater contraceptive use (average probability of 66.9%, CI 95%: 0.65–0.69) than women in urban areas (average predicted probability of 64.3%, CI 95%: 0.63–0.65).

We expand this section by running two additional models using the Pearl Index based on highly efficient methods. Model 7 (Table A4 in the Appendix) shows the regression of the use of highly efficacious methods (Pearl Index < = 0.3) among all sexually active women who reported intending to avoid a pregnancy. Results show that women who have completed at most primary-level studies are significantly less likely to use highly efficient birth control methods (average predicted probability of 24%, CI 95%: 0.22–0.26), as opposed to less efficient methods or no contraception at all, than women with university-level education (average predicted probability of 28.4%, CI 95%: 0.27–0.30). The use of such methods, which encompass hormonal contraception and vasectomy, tends to decline from age 30 with respect to ages 18–24, even when respondents reportedly do not wish to conceive. The average predicted probability of using highly effective contraception at age 18–24 amounts to 45% (CI 95%: 41–48), which can be compared, for instance, to the 34.2% figure corresponding to the 30–34 age category (CI 95%: 0.31–0.37) and the 18.7% probability corresponding to women aged 45+ (CI 95%: 0.17–0.20).

Being single entails a lower average predicted probability of highly effective contraceptive use (23.1%; CI 95%: 0.21–0.25) than being married (31.3%, CI 95%: 0.30–0.33). Practicing a religion is also associated with a lower likelihood of resorting to the noted contraceptive alternatives (average predicted probability of 24.5%. CI 95%: 0.22–0.27) as opposed to being non-practicing (29.4%, CI 95%: 0.29–0.30), while being employed is associated with greater use (average predicted probability of 30.1%, CI 95%: 0.29–0.31) than being non-employed (26.8%, CI 95%: 0.25–0.28). Again, no statistically significant association is found regarding income. In this case, there are no substantial differences between the behavior of foreign-born and Spanish-born women either. When the same model is performed only on women reportedly using contraception (Model 8, see table A4 in the Appendix), most results are maintained. Nevertheless, the educational gradient in the use of effective contraception disappears –women with medium-level studies seem more prone to resort to such alternatives than women with university-level education – as well as the statistical significance of employment and religious practice. Women between 25 and 29, in turn, show greater propensity to use such methods than women under 25.

## Discussion

In this study, we have explored how different aspects of reproductive health such as unintended pregnancies, abortion, and contraceptive use are associated with socioeconomic disadvantage among women in Spain using a national representative survey from 2018. Our results indicate that reproductive choices and outcomes related to unintended pregnancies over the past decades have varied significantly by individuals’ socioeconomic and demographic characteristics along a continuum of social vulnerability. Our first hypothesis—that women in the most vulnerable social positions would be more likely to experience unintended pregnancies and births resulting from unintended pregnancies—has been confirmed. Women with non-university education–especially those with primary-level education or less, those with a migrant background, and those with a history of partnership and employment instability are significantly more likely to have experienced unintended pregnancies. These results align with earlier literature on the topic [1–3, 12]. The probability of a first birth resulting from an unintended pregnancy has been highest among very young women (aged 18 to 24 at birth), those of migrant origin, and those without a partner. Our results, based on large-scale, nationally representative data, confirm that unintended fertility in Spain is strongly linked to social disadvantages, replicating the pattern observed in previous studies [28, 29].

The analysis of abortion incidence yields a mirror image of the patterns observed above. Its prevalence is highest among foreign-born women and those with low to medium levels of education, aligning with our expectations from Hypothesis 2 and recent findings from population-based data [38]. Notably, the positive association between abortion and employment may indicate a certain socioeconomic threshold in use, as we also anticipated. Employed women facing unintended pregnancies, particularly those in precarious labor market conditions, may resort to abortion more frequently to avoid job loss or due to financial constraints and challenges in balancing work and family responsibilities. This interpretation is reinforced by the observed link between employment instability (proxied by a higher number of jobs over time) and abortion. Overall, these findings tentatively support our second hypothesis that relatively disadvantaged women, who are not among the most vulnerable groups (e.g., those without a job), are the most likely to terminate their pregnancies. This is consistent with prior research showing that individuals in more vulnerable situations are more likely to carry unintended pregnancies to term [19]. As expected, union instability also emerges as a key factor associated with the probability of having undergone an abortion. Additionally, cultural and attitudinal factors likely contribute to the lower likelihood of abortion among women who practice religion, as religiosity is a well-established predictor of more restrictive views on abortion [59].

Our third hypothesis proposed a possible explanation for these phenomena –namely, a positive socioeconomic gradient in contraceptive use as an underlying mechanism. This expectation seems corroborated by our findings. Women with university-level education consistently use contraceptive methods when they do not wish to conceive, whereas those with medium-level education, and especially those with only primary-level education, are significantly less likely to do so. However, this educational gradient does not appear to extend so clearly to the choice between highly effective contraception and less effective alternatives or no contraception at all. Among women who use some form of contraception, no significant differences emerge between those at the highest and lowest educational levels. Instead, women with secondary or post-secondary non-university education show greater adherence to highly effective methods, such as hormonal methods and vasectomy, than women with higher education. Women of migrant origin are generally less likely than native-born women to prevent unintended pregnancies through contraception, though no statistically significant differences are found regarding the efficacy of the methods used. Overall, our results indicate that individuals in relatively disadvantaged socioeconomic positions face an increased risk of unintended fertility and abortion primarily due to lower rates of contraceptive use rather than by the selection of less effective methods. Additionally, employment status is positively associated with the use of highly effective contraception, possibly reflecting work-family trade-offs in the labor market. However, financial conditions do not appear to play a significant role. Interestingly, both contraceptive use and reliance on highly effective methods decline steadily after age 30. This trend could be linked to ambivalence towards motherhood at certain ages or to a perception of lower risk of pregnancy among women approaching the end of their reproductive years. Finally, some of the population groups that are most likely to have lower contraceptive use and to have experienced unintended pregnancy (women without university-level education and those with migrant background) are also most prone to abortion. In sum, the fact that, overall, only 64% of women who did not want to conceive used contraception, and only 28.7% used highly effective methods, highlight substantial gaps in effective contraceptive coverage, underscoring persistent vulnerabilities to unintended pregnancy and the need for improved access to highly effective methods.

### Study limitations and future research

This study also entails some limitations. First, although it seems reasonable to expect a linkage between lower contraceptive use among individuals in relatively disadvantaged population strata, and the higher unintended fertility and abortion rates observed within the same groups, the dataset does not permit a causal analysis. It is namely marked by observation time lags – while the use of contraception is assessed at the time of the survey, unintended pregnancies (for the most part) and abortion are measured retrospectively.

In addition, as noted in the methods section, the abortion measure in the 2018 Spanish Fertility Survey likely underestimates abortion prevalence in Spain. The survey relied on indirect questions to distinguish abortions from miscarriages, an approach likely intended to mitigate underreporting due to stigma often associated with abortion. Previous research has clearly shown that abortion is often underreported in survey data [60, 61]. Future research should develop more effective and direct survey measures that accurately capture abortion experiences while remaining sensitive to respondents’ comfort and privacy.

Finally, our study does not include men’s reproductive health experiences. This is in part due to a limited sample of men and due to the common omission of detailed questions on men’s reproductive health in surveys, such as contraceptive use history, involvement in unintended pregnancies and births (when known), or their role in a partner’s (or sexual partner’s) abortion (when known). Men’s reproductive health should be more consistently explored in future research.

## Conclusions

This research represents an important contribution to the literature on reproductive health inequalities, particularly in the Spanish context, where data availability was a limiting factor until the release of the 2018 Fertility Survey. Our findings ultimately highlight the need for reflection on contraceptive accessibility in Spain, which emerges as a key factor for preventing unintended pregnancies and, consequently, abortion. Given that economic conditions –especially income–do not show a significant association with contraceptive use, it is reasonable to assume that other factors are more important. Since individuals with non-university education – especially those with only primary-level studies or less – and those of migrant origin are less likely to use contraceptive methods than highly educated and Spanish-born individuals, limited knowledge of the family-planning system and low awareness of available contraceptive options appear to be the primary challenges to address in reducing unintended pregnancies for the most vulnerable subgroups. These findings are in line with prior studies indicating that lack of contraceptive knowledge is more prevalent among women with lower levels of education [62] and among women of migrant origin [63, 64].

These findings entail some important policy-related implications. Public expenditure should prioritize programs on health education and information campaigns to increase awareness of contraceptive methods, particularly among lower-educated and migrant populations. Additionally, expanding community-based reproductive health services and improving the visibility and accessibility of existing programs (especially in underserved areas) can help reduce structural and informational barriers. For example, primary care settings should reinforce their family-planning counseling and programs with a focus on providing linguistically and culturally adequate services. These public health approaches are important to reduce reproductive health inequalities and improve reproductive outcomes across all social groups.

## Supplementary Information

Supplementary Material 1.

## Data Availability

The data that support the findings of the study (the Spanish Fertility Survey / Encuesta de Fecundidad 2018) are publicly and freely available on the website of the Spanish National Statistics Institute (INE): [https://www.ine.es/dyngs/INEbase/es/operacion.htm?c=Estadistica_C&cid=1254736177006&menu=resultados&idp=1254735573002#_tabs-1254736195425] (section “Microdatos”).

## Footnotes

1 We are aware that women with migrant background are by no means a homogeneous group in terms of socioeconomic status or capacity to access information. Nevertheless, we hypothesize that they could be at a disadvantage with respect to Spanish-born women on several grounds: more limited opportunities to know how to navigate the healthcare/family-planning system, potential accessibility barriers due to language or to narrower, more reduced social support networks in the receiving country, and, during some periods – 2012–2018, see [7] – limited access to healthcare for certain groups (undocumented migrants).

2 All the variables included are either frequent indicators of socioeconomic position (educational-level, migrant origin, employment instability, employment status, income) in the social stratification literature (see, for instance [51], or otherwise potentially associated with reproductive behaviors and outcomes (partnership trajectories, religiosity, rural residence, birth cohort; see [52, 53].

3 The number of co-residential unions is measured up to the survey interview (just as the experience of unintended pregnancy or abortion is considered at any time during the respondents’ reproductive period covered by the survey), since the Spanish Fertility Survey does not permit to identify exactly when a past unintended pregnancy or an abortion took place. The inclusion of a measure of partnership instability in the analysis is important, since the phenomenon could be associated with less predictable sexual activity and thus with less consistent contraceptive planning and use (see f.i [54]), For the same reason, it could also entail a higher probability of abortion. Furthermore, relationship instability itself (and the conflict and challenges often associated with it) has also been documented as one of the most prominent reasons women give for interrupting a pregnancy [55, 56].

4 This variable is also measured up to the time of the survey interview. Employment instability could favor unintended pregnancy by making it economically difficult to access contraception. Additionally, it can be seen as a proxy for a precarious labor market situation, which could increase the likelihood of abortion due to economic difficulties to raise a child, foreseen work-family balance challenges, and high opportunity costs of childbearing.

5 Religiosity has been observed to shape individual attitudes towards contraception and abortion in different societies, even though results are mixed and context-dependent (see for example [52, 57]. In Spain specifically, despite increased societal adherence to secularism, individuals actively practicing Catholicism (and other faiths that similarly do not support sexual activity outside marriage, contraception or abortion) could still be comparatively less likely to use contraceptives or to interrupt a pregnancy.

6 Note that each figure has a different scale due to each outcome’s showing different baseline probabilities.

7 We adjust for age at first birth since it could be a relevant predictor of whether a (first) birth resulted from an unplanned pregnancy (in particular, women who experienced their first birth at a very young age could be more likely to have done so through after an unplanned conception). The woman’s birth cohort is not included as a control simultaneously to avoid multicollinearity concerns (birth cohort is very likely to be related to age at first birth in Spain) and to maintain consistency in the construction of this model, since all covariates and controls are measured at the time (year) of the first birth.

8 Ideally, we would additionally have included a control variable capturing whether the outcome occurred before or after 2010, which is the year when the Organic Law 2/2010 on Sexual and Reproductive Health and the Voluntary interruption of Pregnancy was passed. Unfortunately, this was not possible, since the dataset does not allow to know exactly when (most) unintended pregnancies and abortions took place.

9 The Pearl Index (PI) measures the number of unintended pregnancies per 100 women per year assuming perfect use of the contraceptive method. We have set the threshold at the highest value associated with hormonal contraception, which is generally considered to show very high efficacy. Combined hormonal contraception, progestogen-only pills, injections and implants, the Levonorgestrel intra-uterine system and vasectomy are classified as highly efficacious and thus given the value 1, as they have Pearl Indices equal to or below 0.3. All the remaining methods (barrier methods, fertility-awareness based methods, copper intrauterine devices, and female sterilization) are given the value 0 as potentially less efficacious methods; see Pollacco and Brincat [58].

10 Women using contraception can be assumed to fall within the three categories. Additionally, the survey allows to identify women who do not use contraception for reasons other than (1) being sexually inactive; (2) being pregnant; (3) desiring children. Put together, and excluding those individuals who declare they are biologically infertile, these groups constitute the “at risk for unintended pregnancy” subsample.

11 This finding is somewhat intriguing, since an unintended pregnancy could be argued to generally entail higher costs for a single woman than for a married woman. A possible explanation for the lower propensity to contraceptive use observed among single women is that some have not considered male condoms – which is the most frequent method used within this group – equivalent to own use of contraception. They may have therefore answered negatively when surveyed on contraceptive use by themselves or their partners, especially if they do not consider having occasional sexual encounters as equivalent to having a partner.
