# Supplementary material for: Socioeconomic and demographic differentials in unintended pregnancies, abortion, and contraceptive use in Spain: insights from the 2018 Fertility Survey
Source: Reprod Health. 2026 Apr 14;23:106. doi: 10.1186/s12978-026-02329-6 (PMC13200501; doi:10.1186/s12978-026-02329-6)
Supplement: Supplementary file 1 — Supplementary Material 1. [file 12978_2026_2329_MOESM1_ESM.docx]

**APPENDIX**

**Table A1.** Logistic regression predicting unintended pregnancy experience from women’s socioeconomic and demographic characteristics (odds ratios).

|  |  | *Robustness check:* |
| --- | --- | --- |
|  | Model 1: Unintended pregnancy (all women) | Model 2: Unintended pregnancy (women who have experienced pregnancy) |
| *Education*  *(Ref: University)* |  |  |
| Secondary+ | 1.66*** (0.10) | 1.41*** (0.09) |
| Primary | 2.00*** (0.17) | 1.40*** (0.13) |
| *Migrant origin*  *(Ref: Spanish-born)* |  |  |
| Foreign-born | 2.30*** (0.16) | 1.91*** (0.15) |
| *Birth cohort*  *(Ref: 1960s)* |  |  |
| 1970s | 0.31*** (0.02) | 0.30 *** (0.02) |
| 1980s | 0.24*** (0.018) | 0.32*** (0.03) |
| 1990s+ | 0.13*** (0.01) | 1.06 (0.16) |
| *No. of unions*  *(Ref: No unions)* |  |  |
| 1 | 2.48*** (0.27) | 0.25*** (0.04) |
| 2 | 4.54*** (0.62) | 0.50*** (0.09) |
| 3+ | 7.93*** (1.58) | 1.24 (0.33) |
| *No. of jobs*  *(Ref: No jobs)* |  |  |
| 1 to 3 | 1.46*** (0.17) | 1.05 (0.14) |
| 4 to 6 | 1.74*** (0,21) | 1.33* (0.18) |
| 7+ | 1.76*** (0.20) | 1.40* (0.18) |
| *Religiosity*  *(Ref: Non-practice)* |  |  |
| Practicing | 1.34*** (0.10) | 1.22* (0.10) |
| *Residence*  *(Ref: Urban)* |  |  |
| Rural | 0.91 (0.06) | 0.92 (0.07) |
| Constant | 0.11*** (0.02) | 2.24*** (0.50) |
|  | n: 11290; Pseudo R2: 0.13 | n: 6692; Pseudo R2: 0.09 |

Data source: Spanish Fertility Survey (Encuesta de Fecundidad), 2018. Women aged 18-55 years old. Robust standard errors within brackets. ***p-value <=0.001; **p-value <=0.01; *p-value <=0.05.

**Table A2.** Logistic regression predicting first births from unintended pregnancy from women’s socioeconomic and demographic characteristics (odds ratios).

|  | Model 3: First birth resulting from an unintended pregnancy (as opposed to an intended one) |
| --- | --- |
| *Education, year of first birth (Ref: University)* |  |
| Secondary+ | 1.05 (0.21) |
| Primary | 0.88 (0.27) |
| *Migrant origin (Ref: Spanish-born)* |  |
| Foreign-born | 2.11*** (0.49) |
| *Age at first birth (Ref:18-24 years old)* |  |
| 25-29 | 0.34*** (0.08) |
| 30-34 | 0.16*** (0.04) |
| 35-39 | 0.19*** (0.06) |
| 40-44 | 0.18*** (0.10) |
| 45+ | 0.60 (0.66) |
| *Had a partner, year of first birth (Ref: Partnerless)* |  |
| Had a partner | 0.45** (0.13) |
| *Employment, year of first birth (Ref: Non-employed)* |  |
| Employed | 0.76 (0.14) |
| *Religiosity (Ref: Non-practicing)* |  |
| Practicing | 1.38 (0.37) |
| *Residence (Ref: Rural)* |  |
| Urban | 0.84 (0.19) |
| Constant | 1.44 (0.55) |
| n: 1312, Pseudo R2: 0.11 |  |

Data source: Spanish Fertility Survey (Encuesta de Fecundidad), 2018. Women aged 18-55 years old. Robust standard errors within brackets. ***p-value <=0.001; **p-value <=0.01; *p-value <=0.05.

**Table A3.** Logistic regression predicting abortion from women’s socioeconomic and demographic characteristics (odds ratios).

|  |  | *Robustness check:* |
| --- | --- | --- |
|  | Model 4: Abortion (all women) | Model 5: Abortion (women who have experienced pregnancy) |
| *Education*  *(Ref: University)* |  |  |
| Secondary+ | 1.33* (0.15) | 1.04 ( 0.12) |
| Primary | 1.51** (0.24) | 0.91 (0.15) |
| *Migrant origin*  *(Ref: Spanish-born)* |  |  |
| Foreign-born | 1.97*** (0.23) | 1.48** (0.19) |
| *Birth cohort*  *(Ref: 1960s)* |  |  |
| 1970s | 1.29* (0.16) | 1.18 (0.15) |
| 1980s | 1.35* (0.19) | 1.62*** (0.23) |
| 1990s+ | 1.56* (0.28) | 8.35*** (1.56) |
| *No. of unions*  *(Ref: No unions)* |  |  |
| 1 | 2.50*** (0.52) | 0.48*** (0.11) |
| 2 | 4.74*** (1.12) | 0.96 (0.24) |
| 3+ | 10.38*** (3.10) | 2.44** (0.76) |
| *No. of jobs*  *(Ref: No jobs)* |  |  |
| 1 to 3 | 1.59 (0.38) | 0.93 (0.22) |
| 4 to 6 | 2.35*** (0.57) | 1.44 (0.35) |
| 7+ | 2.44*** (0.58) | 1.55 (0.37) |
| *Religiosity*  *(Ref: Non-practice)* |  |  |
| Practicing | 0.69* (0.11) | 0.58** (0.10) |
| *Residence*  *(Ref: Urban)* |  |  |
| Rural | 0.68** (0.10) | 0.68** (0.10) |
| Constant | 0.01*** (0.001) | 0.07*** (0.02) |
|  | n: 13129; Pseudo R2: 0.05 | n: 7824; Pseudo R2: 0.07 |

Data source: Spanish Fertility Survey (Encuesta de Fecundidad), 2018. Women aged 18-55 years old. Robust standard errors within brackets. ***p-value <=0.001; **p-value <=0.01; *p-value <=0.05.

**Table A4.** Logistic regression predicting contraceptive use from women’s socioeconomic and demographic characteristics (odds ratios).

|  | Model 6: Contraceptive use  (sexually active women not wanting to conceive) | Model 7: Method with Pearl Index >= 0.3 (sexually active women not wanting to conceive) | Model 8: Method with Pearl Index >= 0.3 (Women using contraception) |
| --- | --- | --- | --- |
| *Education (Ref: University)* |  |  |  |
| Secondary+ | 0.71*** (0.04) | 1.09 ( 0.06) | 1.31*** (0.07) |
| Primary | 0.51*** (0.04) | 0.79*** (0.07) | 1.10 (0.10) |
| *Migrant origin (Ref: Spanish-born)* |  |  |  |
| Foreign-born | 0.80*** (0.05) | 0.96 (0.07) | 1.08 (0.09) |
| *Age (Ref: 18-24)* |  |  |  |
| 25-29 years old | 0.83 (0.10) | 1.08 (0.10) | 1.23* (0.12) |
| 30-34 years old | 0.52*** (0.06) | 0.64*** (0.06) | 0.80* (0.08) |
| 35-39 years old | 0.45*** (0.05) | 0.51*** (0.05) | 0.64*** (0.07) |
| 40-44 years old | 0.34*** (0.03) | 0.48*** (0.04) | 0.69*** (0.07) |
| 45+ years old | 0.13*** (0.01) | 0.28*** (0.02) | 0.69*** (0.07) |
| *Partnership status (Ref: Married)* |  |  |  |
| Registered partnership | 1.17 (0.19) | 0.88 (0.14) | 0.81 (0.14) |
| Non-registered cohabitation | 0.88 (0.07) | 0.87 (0.07) | 0.91 (0.08) |
| Non- co-residential relationship | 1.10 (0.09) | 0.94 (0.07) | 0.95 (0.08) |
| Single | 0.52*** (0.03) | 0.64*** (0.04) | 0.83* (0.06) |
| *Employment (Ref: Not employed)* |  |  |  |
| Employed | 1.19** (0.08) | 1.19** (0.08) | 1.11 (0.08) |
| *Own net income (Ref: No income)* |  |  |  |
| <500-999 EUR | 0.98 (0.07) | 0.95 (0.07) | 0.97 (0.08) |
| 1000-1999 EUR | 1.08 (0.09) | 1.06 (0.09) | 1.05 (0.10) |
| 2000-2999 EUR | 1.02 (0.13) | 0.95 (0.13) | 0.92 (0.13) |
| >3000 EUR | 0.81 (0.17) | 0.85 (0.20) | 0.92 (0.25) |
| *Religiosity (Ref: Non-practice)* |  |  |  |
| Practicing | 0.74*** (0.05) | 0.77*** (0.06) | 0.86 (0.07) |
| *Residence (Ref: Urban)* |  |  |  |
| Rural | 1.15* (0.07) | 0.99 (0.06) | 0.93 (0.06) |
| Constant | 8.90*** (0.92) | 0.83* (0.08) | 0.87 (0.09) |
|  | n: 11077  Pseudo R2: 0.11 | n: 11077  Pseudo R2: 0.04 | B: 7172  Pseudo R2: 0.01 |

Data source: Spanish Fertility Survey (Encuesta de Fecundidad), 2018. Women aged 18-55 years old. Robust standard errors within brackets. ***p-value <=0.001; **p-value <=0.01; *p-value <=0.05.
